# Patient Reported Outcomes: misinference from ordinal scales?

**DOI:** 10.1186/1745-6215-12-S1-A65

**Published:** 2011-12-13

**Authors:** Mike Horton, Alan Tennant

**Affiliations:** 1 Psychometric Laboratory for Health Sciences, Department of Rehabilitation Medicine, The University of Leeds, UK

## 

Patient Reported Outcomes (PROs) are widely used in medical outcome studies, and usually take the form of administered of self-completed questionnaires. The data that these questionnaires produce is of the type known as ordinal scaling, where magnitudes of the attribute may be ascertained. At the same time, most outcome studies rely on the calculation of means, standard deviations, change scores, and concepts such as Minimally Important Difference (MID) or effect sizes. Yet, ordinal scales do not support the mathematical operations needed to calculate these type of statistic [3]. Indeed when several items are measured on an ordinal scale it is far from certain that the sum of scores has even ordinal properties [1]. Despite these constraints, these limitations are largely ignored, and thus statistics such as means and MID are widely reported for PROs. This runs the risk of drawing an incorrect inference from data based upon PROs [5].

This risk can be illustrated by considering the concepts of the ‘plateau’ and the calculation of the MID. Both are investigated by contrast of the ordinal raw score against the cardinal metric derived from fit of data to the Rasch measurement model [4]. It can be shown that as the raw score from a scale moves towards the margins, then a smaller and smaller raw score change is associated with a standard metric unit of change. Thus patients may seem to be ‘slowing down’ in their improvement, or even ‘plateauing’, yet they are still moving the same metric distance. Likewise, when considering a magnitude of improvement such as an MID, the raw score distance associated with the MID can be shown to vary across the scale, depending upon the starting point. Thus for one patient the same MID may involve a change in the metric distance four times greater than that of another patient.

PROs provide ordinal estimates of the magnitude of a patient on the trait being measured. Appropriate non-parametric statistics should be used. Else, where possible, the data should be converted to the cardinal metric through use of the Rasch model, which is consistent with the requirements of the theory of Additive Conjoint Measurement [2][6].
